# In vitro selection of DNA aptamers against staphylococcal enterotoxin A

**DOI:** 10.1038/s41598-024-61094-3

**Published:** 2024-05-18

**Authors:** Ricardo Oliveira, Eva Pinho, Maria Margarida Barros, Nuno Filipe Azevedo, Carina Almeida

**Affiliations:** 1 INIAV – National Institute for Agrarian and Veterinarian Research, Rua dos Lagidos, Vairão, 4485-655 Vila do Conde, Portugal; 2 LEPABE – Laboratory for Process Engineering, Environment, Biotechnology and Energy, Faculty of Engineering, University of Porto, Rua Dr. Roberto Frias, 4200-465 Porto, Portugal; 3 AliCE – Associate Laboratory in Chemical Engineering, Faculty of Engineering, University of Porto, Rua Dr. Roberto Frias, 4200-465 Porto, Portugal; 4 Centre of Biological Engineering (CEB), University of Minho, Campus de Gualtar, 4710-057 Braga, Portugal

**Keywords:** SEA, Aptamers, SELEX, Lateral flow assay, Synthetic biology, Evolutionary developmental biology

## Abstract

Staphylococcal enterotoxin A (SEA) is the most frequently reported in staphylococcal food poisoning (SFP) outbreaks. Aptamers are single-stranded nucleic acids that are seen as promising alternatives to antibodies in several areas, including diagnostics. In this work, systematic evolution of ligands by exponential enrichment (SELEX) was used to select DNA aptamers against SEA. The SELEX protocol employed magnetic beads as an immobilization matrix for the target molecule and real-time quantitative PCR (qPCR) for monitoring and optimizing sequence enrichment. After 10 selection cycles, the ssDNA pool with the highest affinity was sequenced by next generation sequencing (NGS). Approximately 3 million aptamer candidates were identified, and the most representative cluster sequences were selected for further characterization. The aptamer with the highest affinity showed an experimental dissociation constant (K_D_) of 13.36 ± 18.62 nM. Increased temperature negatively affected the affinity of the aptamer for the target. Application of the selected aptamers in a lateral flow assay demonstrated their functionality in detecting samples containing 100 ng SEA, the minimum amount capable of causing food poisoning. Overall, the applicability of DNA aptamers in SEA recognition was demonstrated and characterized under different conditions, paving the way for the development of diagnostic tools.

## Introduction

Foodborne pathogens cause approximately 600 million diseases and 420,000 deaths worldwide each year^1,2^. According to the latest EFSA/ECDC report, bacterial toxins are the second most significant cause of foodborne outbreaks (FBO) in Europe^3^. In the United States, *Staphylococcus aureus* and their toxins are also a significant cause of foodborne diseases, causing an estimated 241,000 illnesses per year^4^. However, these numbers probably underestimate the true extent of the problem, as only a fraction of the diseases is properly diagnosed and reported due to lack of routine surveillance of clinical stool specimens, misdiagnosis, self-limiting symptoms, and unreported minor outbreaks and isolated cases. Beside the negative effects on human health, foodborne diseases also cause significant economic losses in lost productivity and related medical expenses^5^. Overall, a large proportion of these consequences could be avoided by adopting preventive food safety measures.

*S. aureus* virulence predominantly relies on the production of a diversity of protein toxins that may have different health implications, including pneumonia, sepsis-related infections, toxic shock syndrome, and food poisoning^6^. Staphylococcal food poisoning (SFP) is usually a result of consumption of food contaminated with pre-formed enterotoxins known as staphylococcal enterotoxins (SEs). SEs are pyrogenic exotoxins, with superantigenic and emetic activity produced by enterotoxigenic *Staphylococcus* species, mainly coagulase-positive staphylococci (CPS). To date, more than 20 SEs and enterotoxin-like proteins (SE-*l*), classified by letters from A to Y have been described (exception to this naming scheme is toxic shock syndrome toxin-1, which was originally named SEF and later renamed because lack emetic activity)^6^. SEA to SEE, also known as classical enterotoxins, are believed to be responsible for approximately 95% of SFP, with SEA being the most frequently reported in SFP events (~ 80%), followed by SED, SEB, SEC, and SEE^6,7^. Ingestion of food products containing as little as 100 ng of these toxins is enough to cause severe disease in immunocompromised patients. For these reasons, SEs are recognized as an important threat for public health, thus their monitoring is essential for food industry^8,9^.

The current reference method for the detection of SEs in foodstuffs (ISO 19020:2017) relies on commercially available polyvalent qualitative enzyme-linked immunoassays (ELISA) with antibodies that detect the classical enterotoxins (SEA-SEE)^10^. However, many limitations and ethical issues are associated with antibodies, as they traditionally require animals for development and production^11^. Alternatively, aptamers have gained importance as a promising alternative to antibodies in several areas, such as diagnosis, therapeutics and drug-delivery^12^. In fact, several aptamers for foodborne pathogens have been described, ranging from whole cells to large proteins, drugs, toxins, small organic molecules or even metal ions^13–23^. Aptamers are short, single-stranded nucleic acids (typically RNA or DNA) that have unique binding characteristics to a target molecule based on a functional three-dimensional structure. Although the functionality is similar, the nature of aptamers makes it possible to overcome some antibodies limitations, such as easier, faster, and cheaper synthesis without the need for animals^11,12^. Aptamers are generated using a repetitive in vitro approach known as Systematic Evolution of Ligands by Exponential Enrichment (SELEX) that starts with a random library of chemically synthesized single-stranded oligonucleotides, which is subjected to consecutive steps of incubation with the target, partitioning, and amplification, until a narrow group of sequences with high affinity and specificity for the target molecule is selected^24^. Although most SELEX procedures follow the principles of these steps, there are several variations of methodologies used to achieve them, for example, different ways of immobilizing the target molecule and separating the binding sequences from the non-binding sequences^25^.

In the present study, the selection of DNA aptamers for SEA is described using a customized SELEX methodology. Streptavidin-magnetic beads were used to immobilize the biotinylated target molecule, while real-time quantitative PCR (qPCR) was used to monitor, optimize, and evaluate the binding affinity of the SEA-binding sequences. Then, next-generation sequencing (NGS) and bioinformatics analysis was applied to identify the most promising aptamers candidates. The combination of these methodologies revealed a simple and fast way to obtain a huge diversity of potential aptamers. SEA served primarily as a model target, but this SELEX procedure may prove crucial in the selection of specific aptamers for a wide variety of molecules of interest, including other food toxins or pathological proteins.

## Results

### Selection of DNA aptamers against SEA

**Figure 1:**
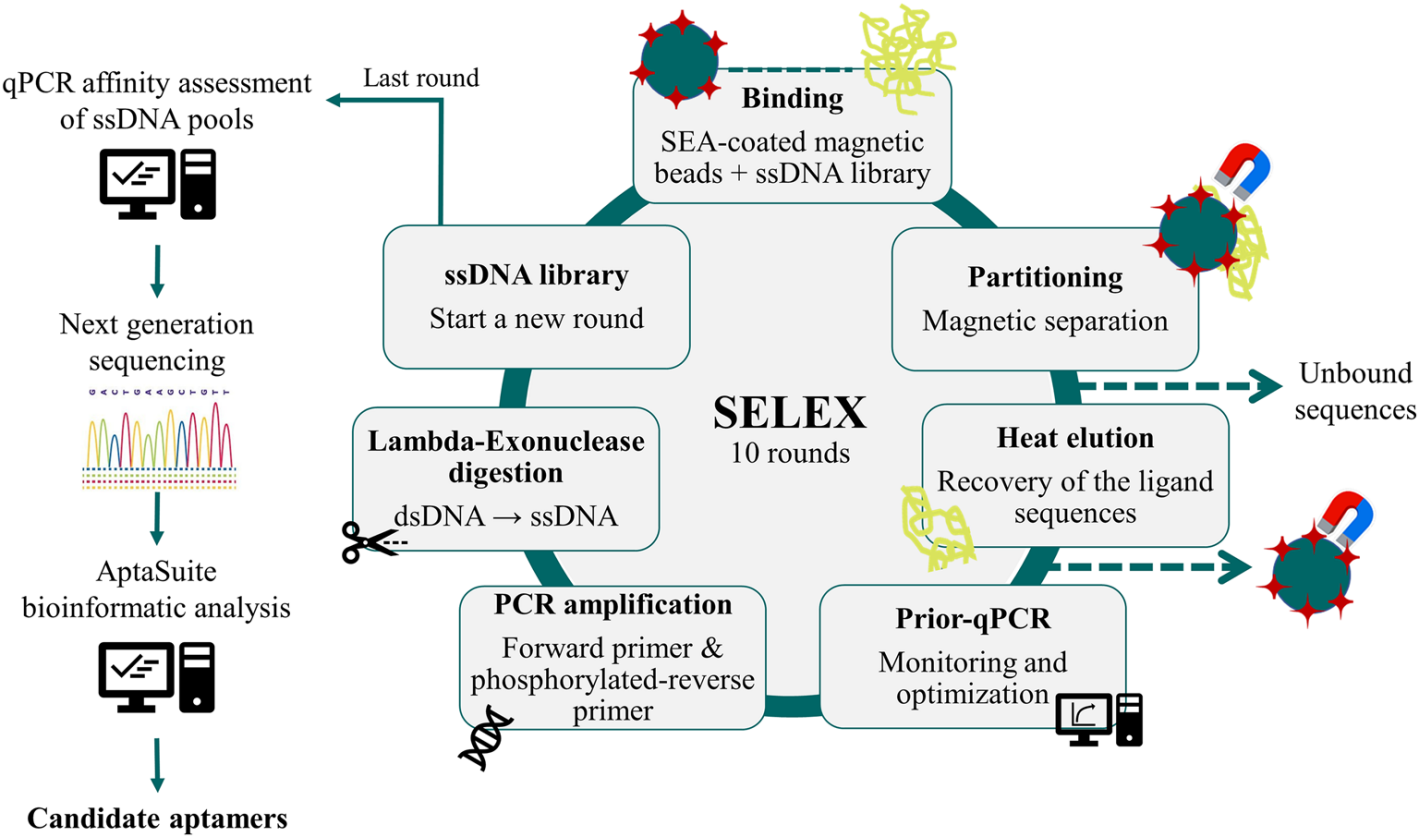
SELEX methodology assembled using streptavidin magnetic beads as an immobilization matrix for the target molecule. In a first step, SEA-coated magnetic beads were incubated with a synthetic library of random sequences under well-defined conditions. Then, SEA-binding sequences were separated by magnetic separation, and the non-binding sequences were discarded. The sequences bound to the immobilized SEA were eluted by heat treatment (95 °C for 10 min). In the amplification step, a qPCR analysis was previously performed to determine the optimal number of PCR cycles for amplification of the ssDNA pool to avoid the formation of nonspecific sequences and/or amplification artifacts. Quantification of the eluted ssDNA pool was also performed to monitor sequence enrichment through the rounds. After that, PCR amplification of the total ssDNA pool was performed using forward primer and phosphorylated-reverse primer according to the optimized PCR cycles. The resulting enriched dsDNA pool was then converted into a new ssDNA pool by Lambda-Exonuclease digestion (selectively digests the 5´-phosphorylated strand of dsDNA) to start a new round of SELEX. These steps were repeated until stabilized enrichment (according to qPCR monitoring) in sequences with affinity for SEA was verified. In the last round, the ssDNA pools from each round were analysed to verify affinity enrichment, and the best ssDNA pool was sent for next generation sequencing (NGS). The raw data was then entered into the AptaSuite software that allowed the identification of all candidate aptamers selected in the process.

In this work, a SELEX protocol using commercial streptavidin-magnetic beads as immobilization matrix for the biotinylated target molecule and qPCR to monitor and optimized the enrichment progress of sequences with affinity to SEA was successful applied. In addition, the methodology applies next-generation sequencing (NGS) along with bioinformatics analysis at the end of the selection process to identify all aptamer candidates (Fig. 1).

The methodology demonstrated the applicability of magnetic beads and qPCR as simple and fast molecular tools for the selection of aptamers (~ 2 weeks). A synthetic ssDNA library consisting of sequences of 97 nt containing a 40 nt randomized core region was used to start the SELEX process and freshly prepared SEA-coated magnetic beads in binding buffer (BB) were used every round. The immobilization efficiency of SEA-coated beads batches prepared for SELEX rounds and binding assays was approximately 2.70 × 10^–6^ pmol SEA/bead and 2.86 × 10^–6^ pmol SEA/bead, respectively. Different incubation conditions (i.e., binding volume, incubation time, negative selection steps, increased wash steps, and addition of competitive agents) were applied throughout the SELEX rounds to strengthen the selection of more specific sequences against SEA. Table 1 summarizes the conditions used in this work for the selection of DNA aptamers against SEA.

**Table 1:** SELEX conditions used in the various steps for the selection of DNA aptamers for SEA.

Round	ssDNA input (pmol)	Binding volume (µL)	Binding time (min)	Negative selection	No. washing steps	Washing Buffer
1	2000	250	30	No	1	Binding buffer (BB)
2	149.5	250	30	No	1	BB
3	174.5	250	30	No	1	BB
4	46.0	100	30	Yes	1	BB
5	52.0	100	30	No	3	BB
6	37.0	100	30	No	3	BB
7	49.5	100	20	No	3	BB
8	36.0	100	20	Yes	3	BB + 0.5 ug/mL bovine serum albumin (BSA)
9	32.5	100	10	No	3	BB + 0.5 ug/mL BSA
10	39.0	100	10	No	3	BB + 0.5 ug/mL BSA

The amount of ssDNA input in each round corresponds to the total amount obtained after Lambda-exonuclease digestion. All SELEX rounds were performed at room temperature (≈ 25 °C) using ≈ 1 × 10^7^ SEA-coated beads (corresponding ≈ 2.70 × 10^–6^ pmol SEA/bead). Negative selection rounds were performed with uncoated streptavidin-magnetic beads.

**Figure 2:**
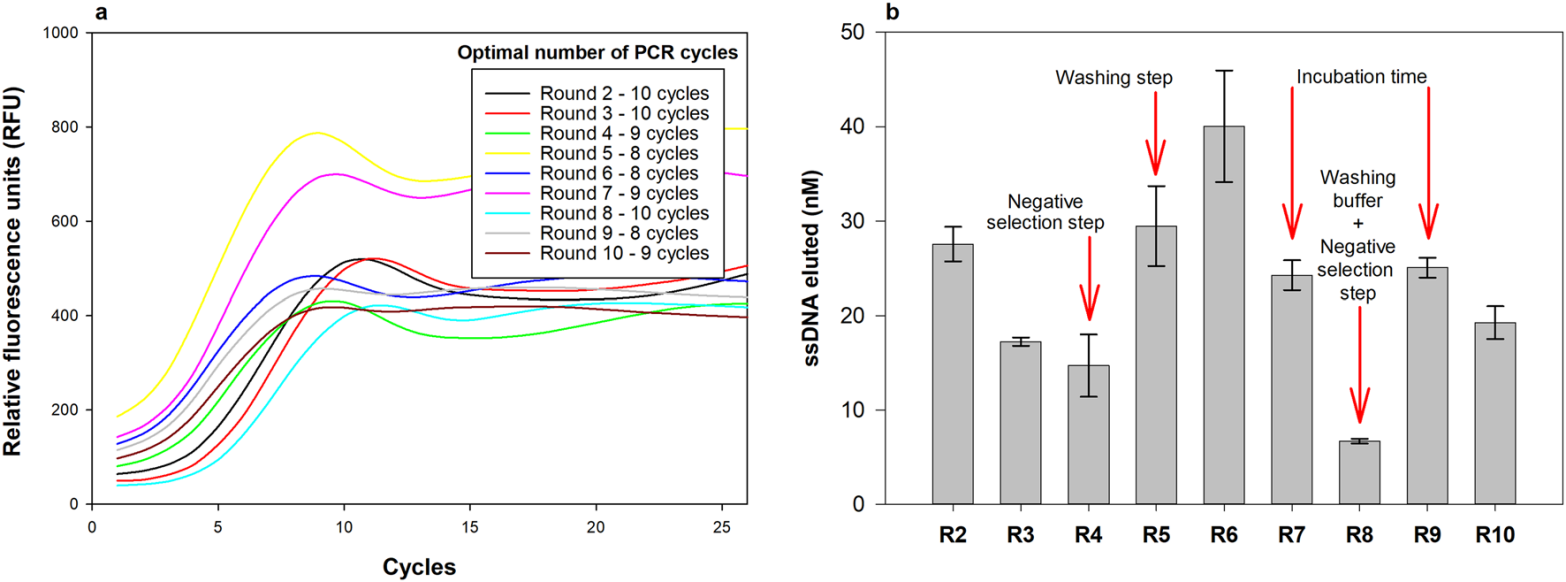
(**a**) Amplification profiles of the prior-qPCR reaction from each round of SELEX used to determine the minimum number of cycles for the PCR amplification step that prevent the formation of by-products and artifacts, i.e., the number of cycles before the amplification curve reaches maximum SYBR green fluorescence. (**b**) Control of the enrichment of the eluted ssDNA pools in each round (R) by qPCR. The arrows highlight the rounds in which different selection conditions (increased stringency) were applied. Duplicates of the reactions as well as a non-target control (NTC) were included in each qPCR assay to ensure the absence of contamination.

The streptavidin-functionalized magnetic beads allowed simple and efficient immobilization of the biotinylated SEA, as well as easy separation system of the binding sequences from non-binding sequences by magnetic separation throughout the selection process. In turn, qPCR allowed for rapid, real-time optimization of the number of PCR cycles for the amplification step in each round, avoiding the formation of by-products and artefacts due to over-amplification and minimizing the PCR bias. The number of optimal cycles determined for each round were in the range of 8–10 cycles (Fig. 2a). Then, the use of the 3′-phosporylated primer during PCR amplification allowed easy conversion of the amplified sequences (dsDNA) into single-stranded sequences by Lambda-exonuclease digestion. The single-stranded sequences were then used to start the new rounds. In round 2 and 8, uncoated magnetic beads were used as negative selection step (before incubation with SEA) to eliminate any sequences that unspecifically bind to the surface of the beads that may not have been completely coated with SEA. For this, the ssDNA pool from the previous round was incubated first with uncoated streptavidin-magnetic beads, and unbound sequences were recovered for the subsequent incubation with the SEA-coated beads.

A total of 10 rounds (R1–R10) of selection were performed. The eluted ssDNA pools (after incubation and partitioning steps) were quantified by qPCR in each round (Fig. 2b). R1 is not shown as the entire volume of eluted ssDNA pool was amplified to recover all binding sequences and, thus, avoid the loss of potential aptamers in the initial phase, usually critical in the selection process^26^.

After an initial stabilization phase (R2–4), an enrichment in sequences (increased concentration of ssDNA eluted) was observed (R4–R6), even increasing the washing steps (Fig. 1b). In turn, the first reduction in incubation time (R7) and the addition of a competitive agent in combination with another negative selection step (R8) had a decreasing effect on the fraction of bound sequences. However, the subsequent reduction of the incubation time (10 min) had no impact in the stringency of the selection (increased amount of ssDNA eluted in round 9). Another round under the same conditions (R10) shown a small variation on sequence enrichment, so that it was estimated that the ssDNA pools were stabilized and that adequate affinity for SEA (Fig. 1b).

**Figure 3:**
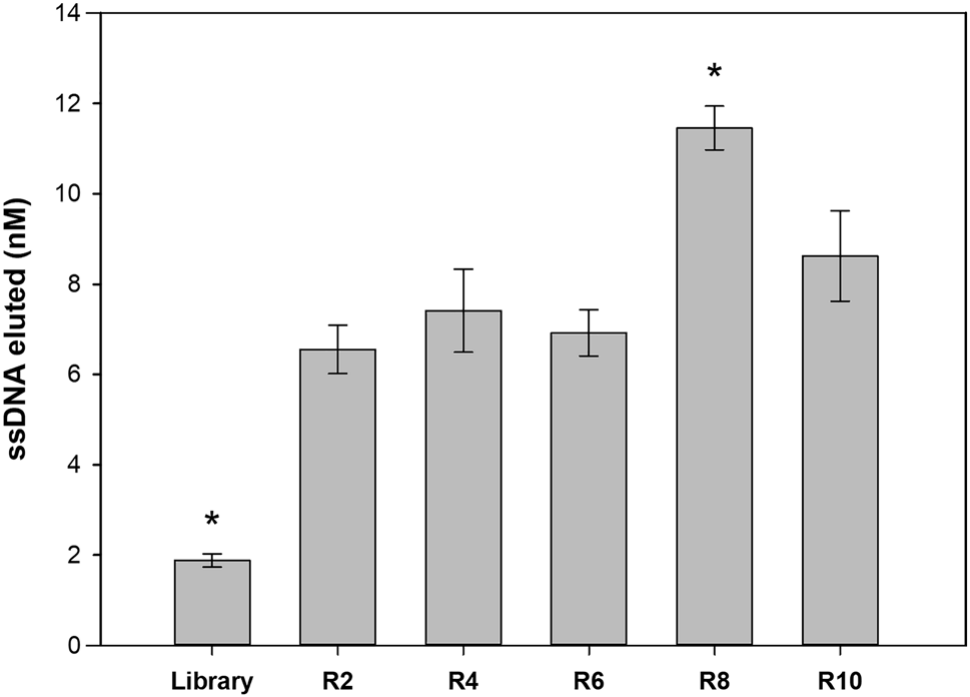
Affinity assay of ssDNA pools (standardized to 125 nM) from rounds 2, 4, 6, 8 and 10 against ≈ 28.6 pmols SEA (10^7^ coated beads) under the same incubation conditions (i.e., 30 min at 25 °C in 100 µL of BB). Duplicates of the reactions as well as an NTC were included in each qPCR assay to ensure the absence of contamination. *ssDNA concentration is statistically different when compared with the other rounds (one-way ANOVA; *P* < 0.05).

At the end, the affinity progression against SEA was evaluated for ssDNA pools selected from R2, R4, R6, R8 and R10 and compared the initial library. For this, ssDNA pools were standardized to 125 nM and incubated under the same incubation conditions with SEA-coated beads. The fraction of bound ssDNA sequences of each round was then quantified by qPCR (Fig. 3).

All ssDNA pools tested showed a significantly higher affinity for SEA compared to the initial library. The affinity of the R2, R4, R6 and R10 was statistically equivalent. However, the affinity determined for R8 was significantly higher than for the other rounds tested. Thus, the ssDNA pool from R8 was determined by next-generation sequencing (NGS).

### Identification of ssDNA aptamer candidates by next-generation sequencing (NGS)

The NGS sequencing raw data from the R8 pool was analysed using the AptaSUITE software. By defining the fixed primer sequences, this software automatically identified all sequences that fit the aptamer properties defined initially (i.e., flanked primer sequences and 40 nt core region). The final sequencing pool had a variety of sequences that did not meet these requirements (e.g., some contained primer zones with point mutations as well as core regions with < or > 40 nt), but these were excluded. A total of 2,914,364 candidate aptamers were identified, from which 2,611,982 (89.62%) were unique sequences and 302,382 (10.38%) had more than one copy. A cluster analysis of sequence similarity was performed, and the sequences were grouped based on their nucleotide sequences. Among the various sequence clusters obtained, the 5 clusters with the highest total counts were selected (Table 2),

**Table 2:** Top 5 clusters and their constituent candidate sequences in terms of total count obtained by AptaSUITE software^27^.

Cluster 1 sequences (5′–3′)	Cluster size
** > Aptamer_301794**	19
AGGCCAACTGGATAGCGAATAGAACTCTGCCGTGGGGTGCAGTTTTAATTGTACGTTCTCGAATTCAGCACTACCTTTTGGCAAACGCTAATAAGGG	
** > Aptamer_1403717**	2
**G**GGCCAACTGGATAGCGAATAGAACTCTGCCGTGGGGTGCAGTTTTAATTGTACGTTCTCGAATTCAGCACTACCTTTTGGCAAACGCTAATAAGGG	
** > Aptamer_2065068**	1
AGGCCAACTGGATAGCGAATAGAACTCTGCCGTGGGGTGCAGTTTTAATTGTACG**C**TCTCGAATTCAGCACTACCTTTTGGCAAACGCTAATAAGGG	
** > Aptamer_458952**	1
**G**GGCCAACTGGATCGCGAATAGAACTCTGCCGTGGGGTGCAGTTTTAATTGTACGTTCTCGAATTCAGCA**T**TACCTTTTGGCAAACGCTAATAAGGG	
** > Aptamer_487442**	1
AGGCCAACTGGAT**C**GCGAATAGAACTCTGCCGTGGGGTGCAGTTTTAATTGTACGTTCTCGAATTCAGCACTACCTTTTGGCAAACGCTAATAAGGG	
**Cluster 2 sequences** (5′–3′)	
** > Aptamer_207452**	16
AGGCCAACTGGATAGCGAATGTGGCAGTCGGTTTTGACTTGCGCTCTTGCATCATTATTCGAATTCAGCACTACCTTTTGGCAAACGCTAATAAGGG	
** > Aptamer_304945**	1
**G**GGCCAAC**C**GGATAGCGAATGTGGCAGTCGGTTTTGACTTGCGCTCTTGCATCATTATTCGAATTCAGCACTACCTTTTGGCAAACGCTAATAAGGG	
** > Aptamer_1441463**	1
AGGCCAACTGGATAGCGAATGTGGCAGTCGGTTTTGACTTGCGCTCTTGCATCA**A**TATTCGAA**A**TCAGCACTACCTTTTGGCAAACGCTAATAAGGG	
** > Aptamer_144392**	1
AGGCCAACTGGATAGCGAATGTGGCAGTCGGTTTTGACTTGCGCTCTTGCATCATTATTCGAATTCAGCA**A**TACCTTTTGGCAAACGCTAATAAGGG	
** > Aptamer_264813**	1
AGGCCAACTGGATAGCGAATGTGGCAGTCGGTTTTGACTTGCGCTCTTGCATCATTATTCGAAT**A**CAGCACTACCTTTTGGCAAACGCTAATAAGGG	
**Cluster 3 sequences** (5′–3′)	
** > Aptamer_546515**	6
AGGCCAACTGGATAGCGAAGTGGGAGAGGAGTGGGGGGTATGGTGCATAAAGTCTGTTGCGAATTCAGCACTACCTTTTGGCAAACGCTAATAAGGG	
** > Aptamer_111056**	2
AGGCCAACTGGATAGCGAAGTGGGAGAGGAGTGGGGGGTATGGTGCATAAAGTCTGTTGCGAATTCAGCACTACCTTTTGGCAAACGCTAATAAGG**A**	
** > Aptamer_947713**	1
**G**GGCCAACTGGATAGCGAAGTGGGAGAGGAGTGGGGGGTATGGTGCATAAAGTCTGTTGCGAATTCAGCACTACCTTTTGGCAAACGCTAATAAGGG	
**Cluster 4 sequences** (5′–3′)	
** > Aptamer_423283**	9
AGGCCAACTGGATAGCGAATGTTAGTGGACTCGGTTTGGACGTTCTCTCTTCTCGAATGCGAATTCAGCACTACCTTTTGGCAAACGCTAATAAGGG	
**Cluster 5 sequences** (5′–3′)	
** > Aptamer_253457**	7
AGGCCAACTGGATAGCGAATTTGCAATCAGCGTGGGTGGGTTTCTTTTGCGTGGTGTGTCGAATTCAGCACTACCTTTTGGCAAACGCTAATAAGGG	
** > Aptamer_2556277**	1
AGGCCAACTGGATAGCGAATTTGCAATCAGCGTGGGTGGGTTTCTTTTGCGTGGT**G**GGTCGAATTCAGCACTACCTTTTGGCAAACGCTAATAAGGG	

The underlined sections represent the fixed primer regions. Nucleotide variations between sequences in the same cluster are highlighted in bold.

**Figure 4:**
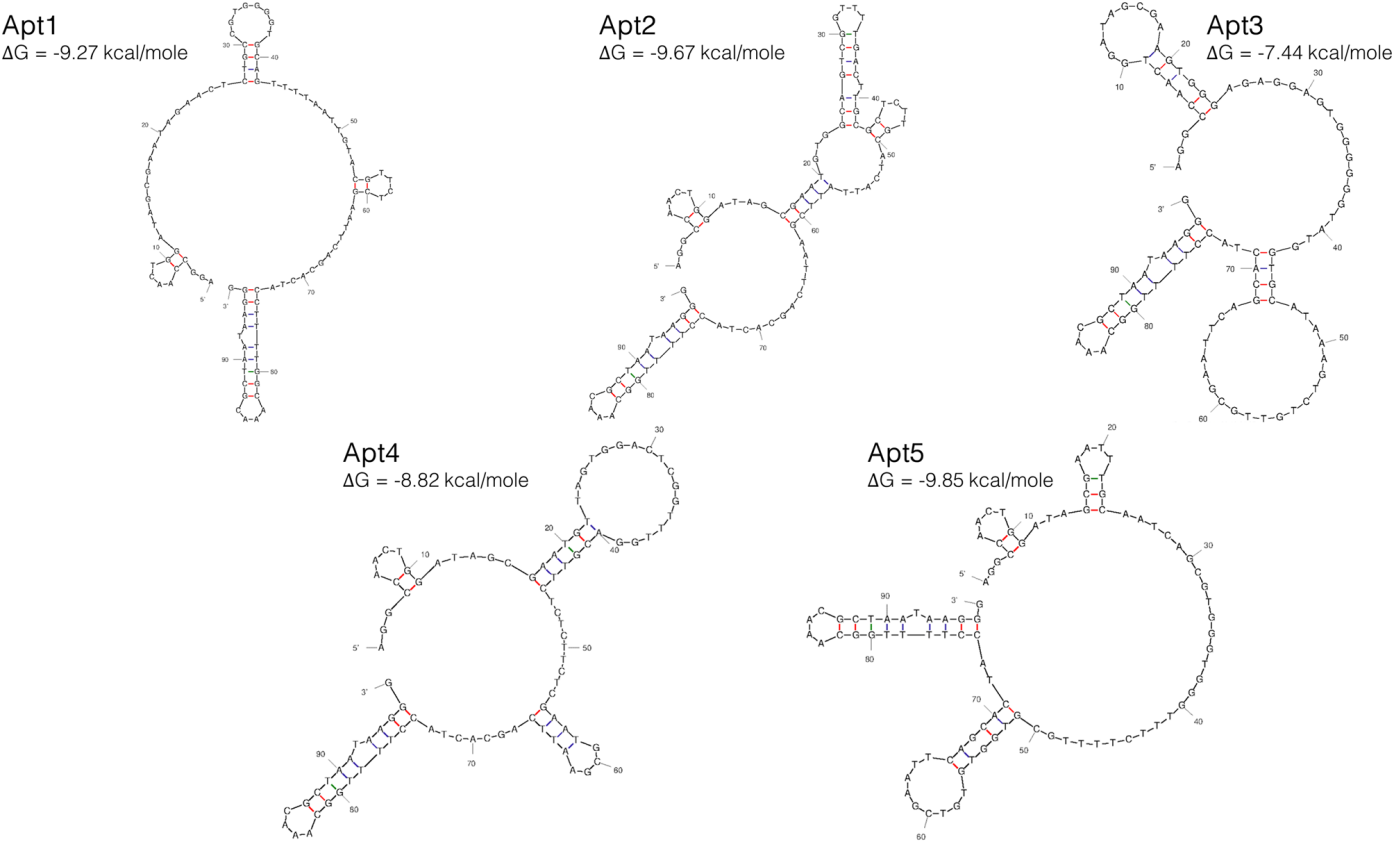
Secondary structures with minimum Gibbs free energies (ΔG) of the sequences representing the top 5 clusters predicted using DNA folding form of the mFold software^28^. The structures were predicted using the following conditions: 25 °C, 138 mM Na^+^ and 0.5 mM Mg^2+^.

The sequences within each cluster show minor variations in nucleotide sequences (one- or two-point nucleotide variations) in both the core region and/or the primer region. The sequence with the highest count within each cluster was selected as representative of the cluster for assessing the affinity for SEA. Based on these criteria, aptamer 301,794 (renamed Apt1), aptamer 207,452 (renamed Apt2), aptamer 546,515 (renamed Apt3), aptamer 423,283 (renamed Apt4) and aptamer 253,457 (renamed Apt5) were synthesized. Their predicted secondary structures and minimal free energies are shown in Fig. 4.

The five aptamers have different secondary structures characterized by various intramolecular structures, including stems, harpin loops, and bulges. Comparing the most thermodynamically favourable structure of each aptamer, all share a structural motif near the 3′ terminus between 74 and 96 nucleotides, while Apt1, Apt2, Apt4 and Apt5 share another structural motif near the 5′ terminus between 4 and 11 nucleotides. Both motifs are in the fixed primer regions. No common structural motif is found in the central random region. Apt5 has the most negative ΔG energy value (− 9.85 kcal/mole), followed by Apt2 (− 9.67 kcal/mole), Apt1 (− 9.27 kcal/mole), Apt4 (− 8.82 kcal/mole), and Apt3 (− 7.44 kcal/mole).

### Aptamer binding affinity and dissociation constant (K_D_)

**Figure 5:**
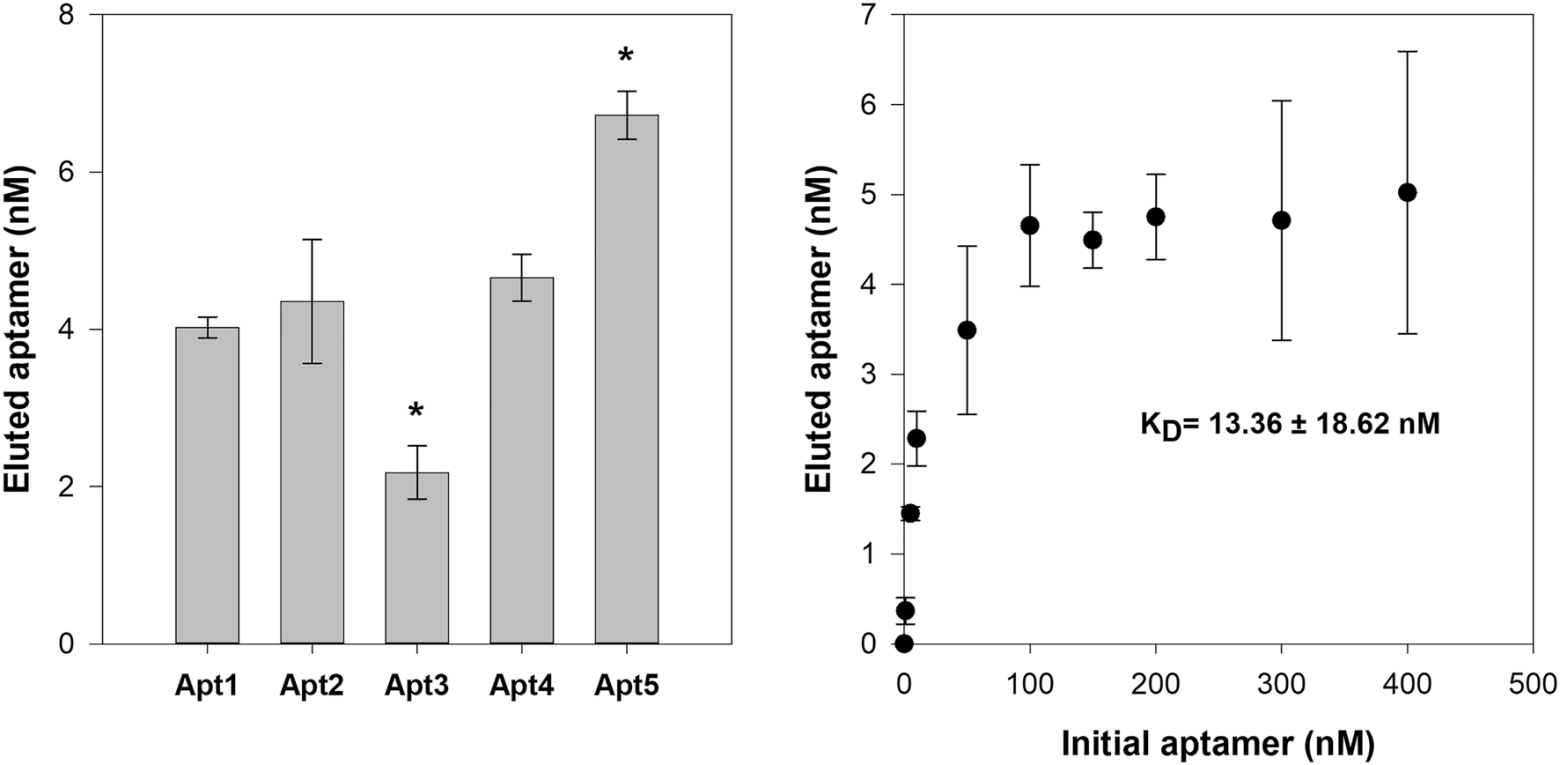
(**a**) Affinity assay of the candidate aptamers (standardized to 125 nM) against ≈28.6 pmols SEA (10^7^ coated beads) under the same incubation conditions (i.e., 30 min at 25 °C in 100 µL of BB). Triplicates of the binding reactions were performed, and duplicates as well as a NTC were included in each qPCR assay to ensure the absence of contamination. *ssDNA concentration is statistically different when compared with the other aptamer candidates (one-way ANOVA; *P* < 0.05). (**b**) Binding saturation curve of Apt5 against SEA. Approximately ≈28.6 pmols SEA (10^7^ coated beads) were incubated with increasing concentrations of aptamer (0, 1, 5, 10, 50, 100, 150, 200, 300 and 400 nM) and the concentration of bound sequences was then measured by qPCR. Data points represent the mean of three replicates. A non-linear regression curve was fitted to the data using SigmaPlot version 12.5 and the dissociation constant (K_D_) is shown as the mean ± standard deviation (SD).

An affinity assay was performed with a standardized concentration (125 nM) of each candidate aptamer against a constant amount of SEA (≈ 28.6 pmols SEA/10^7^ coated beads) under the same conditions (i.e., 30 min at 25 °C in 100 µL of BB). After binding, the amount of bound aptamer was quantified by qPCR (Fig. 5a). Based on the affinity assay, Apt1, Apt2 and Apt4 showed equivalent affinity for SEA, while Apt3 showed a significantly lower affinity. Apt5 was found to have a significantly higher affinity than the other candidates and was selected for further characterization. The dissociation constant (K_D_) was determined using a range of concentrations (1–400 nM) of aptamer against a constant amount of SEA (≈ 28.6 pmols SEA/10^7^ coated beads) under the same conditions used previously in the affinity assays. The concentration of SEA-bound Apt5 was calculated by qPCR and a non-linear regression curve was fitted to the data. The K_D_ obtained was 13.36 ± 1.86 nM (Fig. 5b).

### Effects of temperature on aptamer binding affinity

**Figure 6:**
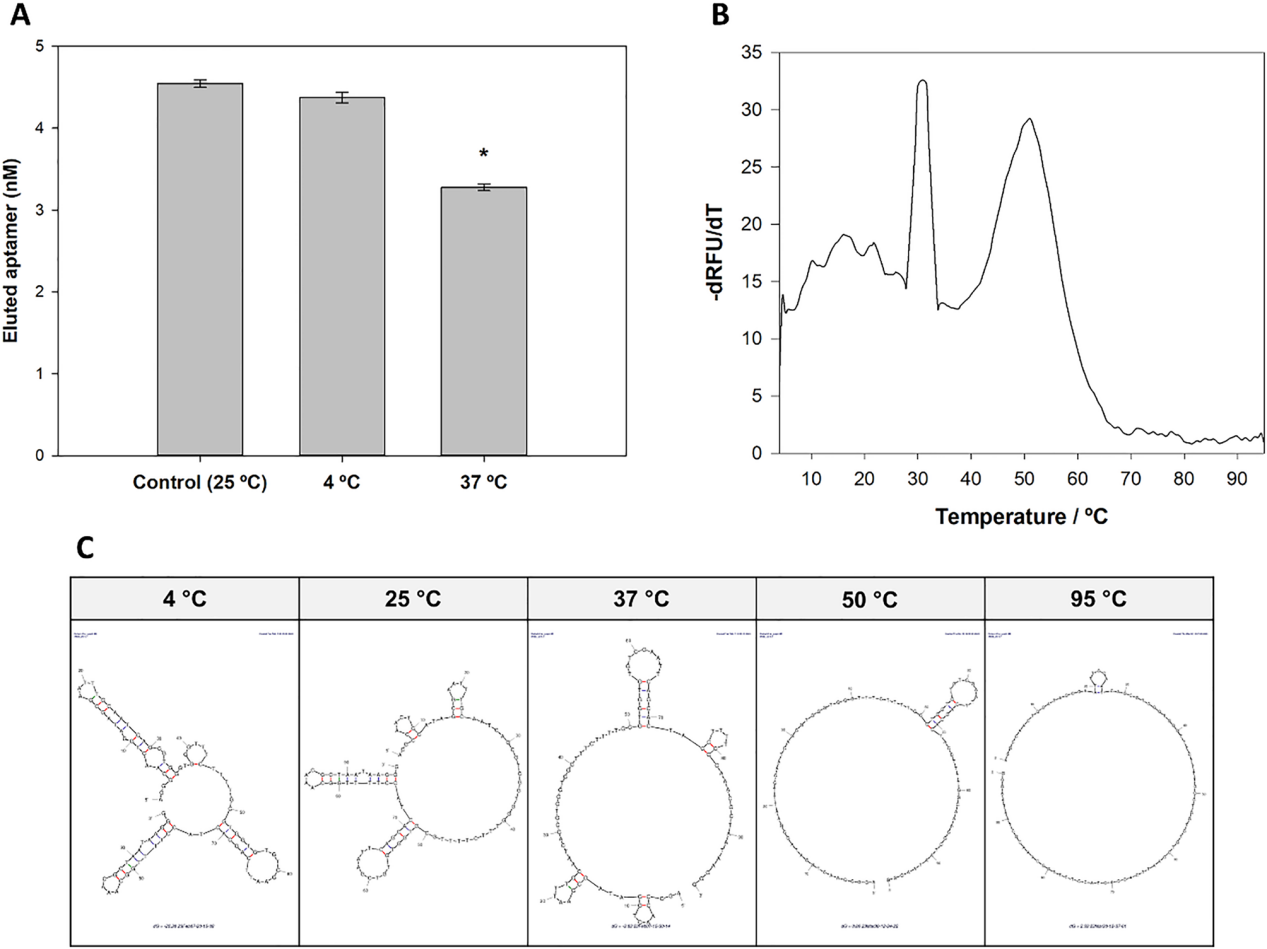
Effect of temperature on Apt5 properties. (**A**) Binding assay of a single concentration (125 nM) of Apt5 at different temperatures (4 °C, 25 °C and 37 °C). Amount of bound aptamer was quantified by qPCR. *ssDNA concentration is statistically different when compared with the other conditions tested (one-way ANOVA; *P* < 0.05). (**B**) Melting curve of Apt5 in binding buffer using SYBR green between 4 °C and 95 °C. (C) Secondary structure prediction of the DNA sequence at 4 °C, 25 °C, 37 °C, 50 °C and 95 °C at 138 mM Na^2+^ and 0.5 mM Mg^2+^ using mfold software.

To anticipate possible variations in the binding temperature of aptamers with the target molecule in the final application, the thermal stability (*i.e.,* effect of temperature) of Apt5 was assessed. The assays were performed similarly to the affinity assays but at 4 °C and 37 °C, and bound aptamer was quantified by qPCR. Data obtained showed that the aptamer binding affinity significantly decreases when the incubation temperature was 37 °C, however at 4 °C there is no significant change compared to original conditions (25 °C) (Fig. 6A). In order to justify these variations in affinity, an analysis of the melting curve of the aptamer in solution was performed. The thermal profile (-dRFU/dT) of Apt5 suggests a pronounced structural change at approximately 30–33 °C and at 45–50 °C by the variation in aptamer fluorescence. Similarly*, *in silico predictions at increasing temperatures also indicate potential changes in the aptamer’s secondary structure, with the progressive opening of the duplex zones (Fig. 6B and C).

### Aptamer-based lateral flow assay

**Figure 7:**
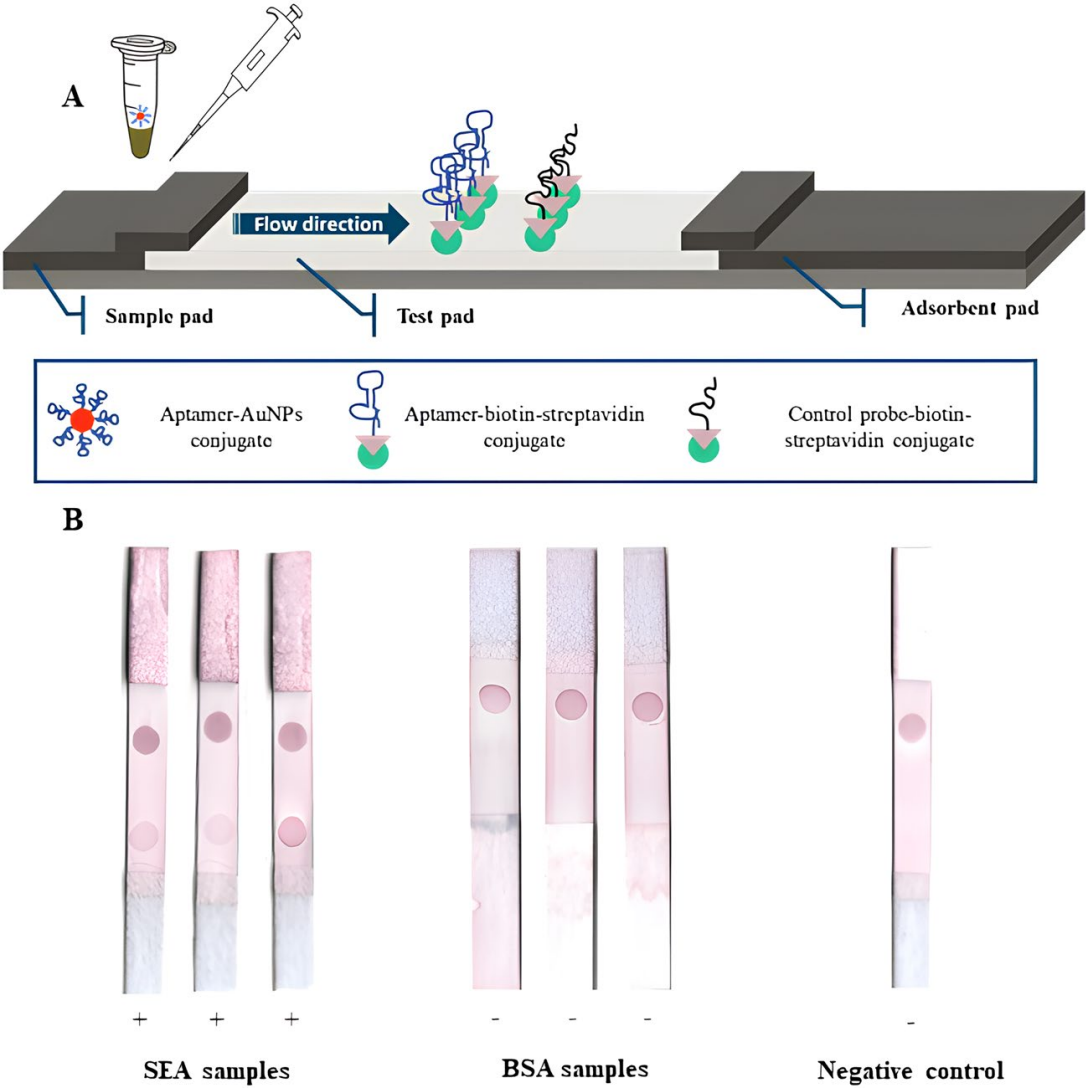
Lateral flow test based on aptamers selected for SEA. (**A**) Schematic of the structure and composition of the assembled LFA assay. (**B**) Proof-of-concept test of SEA samples (100 ng) as well as BSA and BB samples on LFA strips assembled on the basis of previous work.

Based on previous work with lateral flow assays (LFA), strips consisting of a sample zone, a test zone and an absorbent zone were assembled with optimized materials (Fig. 7A). The synthesis of AuNPs functionalized with Apt5 was achieved commercially, as was the biotinylation of Apt1, Apt2, Apt3 and Apt4. Immobilization of the biotinylated aptamers on the test zone of the nitrocellulose membrane was achieved using streptavidin as an anchor, as has been well described in other studies. Immobilization of a probe complementary to the 5′ end of Apt5 in the control zone was used as a test control. The addition of 0.01% tween-20 to BB was based on previous results in other studies, since without a surfactant the sample is less able to move on the test membrane. The interference of tween-20 in the binding of the aptamer to the target molecule was evaluated, and it was found that it does not have a negative effect (data not shown). The prepared mixture containing 100 ng of SEA, Apt5-AuNPs (OD3) and BB with 0.01% tween-20 was incubated under the selection conditions and then applied to the sample membrane. After 10 min, AuNPs could be seen accumulating in the test zone, revealing a positive result. Negative samples containing only BB and BSA showed no result in the test zone. All of them showed accumulation of AuNPs in the control zone, validating the test (Fig. 7B).

## Discussion

Aptamers have proven to be a promising molecular tool in several areas from diagnostics to therapy^19,29–31^. The simple, inexpensive, and fast synthesis of nucleic acids are the main advantages over their functional counterparts. Furthermore, traditional antibodies usually require animals for development, although some animal-free techniques (e.g., phage display) have been explored^11^. In turn, aptamers are selected through an in vitro technique allowing the use of any target molecule, including highly toxic compounds that could kill the animals in a traditional antibody development^32,33^. In food safety, aptamer-based assays have been developed for several food contaminants such as microorganisms, viruses, heavy metal ions, biotoxins, adulterants, dyes, antibiotics, pesticides, and fertilizers^33–36^. Their application to the treatment of specific medical conditions has also been reported and, in fact, some solutions have reached the market^37,38^.

Aptamers for SEA have already been selected based on different SELEX methodologies that involves binding, partitioning, amplification, and conditioning steps using techniques that provide little information on the enrichment of sequences and/or chemical procedures, such as the preparation of magnetic nanoparticles, which require some prior knowledge and experience. Final pools were also used for cloning and sanger sequencing to identify potential aptamers^21,39^. All these limitations then reflect the inability of other researchers to apply SELEX methodologies in their laboratories and/or the low probability of finding good aptamers. Currently, high-throughput molecular techniques and bioinformatic tools have evolved to a new level and allow a more detailed evaluation of the sets of enriched sequences, so that a tailored selection of aptamers candidates can be made, increasing the odds of finding aptamers with suitable properties. Also, little is known regarding the stability of the described aptamers under different conditions, which could be relevant information for the final application of aptamers in diagnostic or even therapeutic solutions.

In this work a SELEX methodology using current molecular techniques for the selection of DNA aptamers was successfully assembled (Fig. 1). Commercially magnetic beads had already demonstrated their enormous applicability in SELEX as well as qPCR^26,40,41^. Streptavidin-biotin immobilization strategy requires small amount of biotinylated target molecules that are often commercially available or easily achievable using biotinylation kits and are an effective way to separate the binding sequences from non-binding sequences, without requiring a complex technique or equipment (e.g., filtration or electrophoresis), making SELEX rounds faster^17,40^. In turn, qPCR is very common in any laboratory nowadays and allows a real-time optimization of the amplification of candidate sequences as well as their quantification. By determining the optimal number of amplification cycles, the amplification of non-specific sequences, artifacts and/or preferential amplification (PCR bias) is avoided. It also allows real-time monitoring of enrichment through quantification against a standard curve (Fig. 2)^15,17,18,21,39,42^. For the characterization of aptamers, qPCR can also be a valuable technique for binding assays (including K_D_ determination), proving to be a simple, fast and inexpensive alternative to specialized techniques (e.g., surface plasmon resonance or isothermal titration calorimetry) or on the introduction of fluorescent labelling in the sequences (e.g., 6-carboxyfluorescein, 6-FAM) (Fig. 3 and 5)^26^.

SEA was used as a study model, since it is the most reported SE involved in foodborne outbreaks, but any foodborne toxins or other protein of interest could be used with this methodology^6,7,43^. Ten rounds of SELEX with increased stringency conditions were adequate to identify pools with higher affinity for SEA compared to the initial library (Fig. 3). Decreasing the incubation time (R7) and adding a competing agent (i.e., BSA) to the washing buffer (R8) were the stringency conditions with the greatest effect on the sequence enrichment process, leading to a significant reduction in the concentration of selected sequences (Fig. 2B). This reduction should be seen as an enrichment of sequences more specific to SEA and the elimination of sequences with lower affinity to SEA due to the increased stringency. This hypothesis is confirmed by the results obtained on the affinity assay of the standardized pools from each round. According to the affinity assays (Fig. 3), the sequences present in R8 showed a higher affinity, comparative to other rounds, despite this round having a low concentration of ssDNA eluted. Thus, this pool should contain a restricted group of very specific SEA sequences and promising candidates for the selection of aptamers with high specificity for SEA.

NGS proved to be an effective tool to identify all possible candidate sequences with affinity for SEA. Traditional cloning and sanger sequencing are based on the selection of a limited number of transformed colonies, so that only a small percentage of the sequences are uncovered. Similar to other studies, NGS allowed a complete view of the ssDNA pools selected in SELEX protocols^26,44^. In addition, NGS results was then analysed by AptaSUITE software, which allowed a simple and fast analysis of the raw data, identifying millions of candidate sequences and grouping them into clusters^27^. The sequences contained in each cluster had minor variations (maximum two nucleotides apart) in the central region and/or the primer region (Table 2). These variations represent a variability of sequences that have similar functional structures and can therefore be considered to contribute the same affinity to the target molecule^45^. Thus, cluster analysis was chosen for the selection of candidate aptamers rather than analysis of individual sequence frequencies, since the clusters contain very similar sequences that most probably contain equivalent SEA binding characteristics^46^. Common structural motifs were identified in the primer fixed regions of the secondary structures of the top 5 aptamers, which could mean a functional role in their affinity for the target molecule (Fig. 4). Still, the various structures present in the random central region of each aptamer should play an important role in stabilizing binding structure to the target molecule^47^. Regarding the ΔG values, energy required to break the secondary structure, Apt5 shows the most negative value, which means that it has the most thermodynamically stable structure, while Apt3 has the least thermodynamically stable structure^48^.

Based on the simple affinity evaluation (Fig. 5A), Apt5 had a greater binding and was therefore characterized in more detail. Data obtained from K_D_ analysis showed a typical saturation curve and fitted by non-linear regression analysis, which indicates that the aptamer interacts with SEA in a concentration-dependent manner (Fig. 5B). The determined K_D_ was 13.36 ± 18.62 nM, which is in the same value range (nM) as most aptamers with application and is better or comparable to other aptamers described for SEA. Wang et al. identified two aptamers (S3 and S12) with K_D_ values of 36.93 ± 7.29 nM and 79.35 ± 12.09 nM, respectively^21^. Huang et al. also identified three aptamers (A1, A15 and A23.2) with K_D_ values of 92.17 ± 16.75 nM, 48.57 ± 6.52 nM and 235.00 ± 79.05 nM, respectively^39^. Furthermore, Apt5 showed a K_D_ in the same range (nM) as SEA-specific antibodies^49,50^. Yet, newly selected antibodies to SEA showed affinity in the pM range^51^. However, the identified aptamers might be further subjected to modifications to achieve equivalent sensitivity^52^.

Since aptamers to be applied in food matrices, or even in point-of-need (PoN) solutions, can find a broad range of conditions, the temperature effect on the Apt5 performance was evaluated. Such factor can significantly affect the affinity of the aptamer to the target molecule since the interaction complex is dependent on the physical fitting between the aptamer and the protein^53^. This study successfully applied melting curve analysis to characterize the thermal stability of aptamers. This methodology, normally used to characterize dsDNA, was successfully used here to characterize the thermal profile of ssDNA, but which, as shown in Fig. 6C, has duplex regions that form the three-dimensional structure of the aptamers. Increasing the temperature to 37 °C significantly affected the affinity to SEA, but the decrease to 4 °C has not significantly influenced the affinity (Fig. 6A). Temperature increase mainly causes denaturation of the intramolecular bonds and change the three-dimensional structure of the aptamers and proteins^54^. Analysis of the Apt5 melting curve (Fig. 6B) shows that, at 31.5 °C and 51 °C, the aptamer shows a fluorescence variation (*i.e.,* -dRFU/dT peak, which results from the decrease in SYBR green fluorescence during dissociation of dsDNA). Aptamers are single-stranded nucleic acids with intramolecular pairing zones that form dsDNA regions, known as stems. These zones are formed based on Watson–Crick base pairing, so their separation during heating generate fluorescence variations^55^. The fluorescence peak at 31.5 °C may be a reason for the loss of affinity verified in the binding assays at 37 °C. For temperatures lower than 31.5 °C, there are small variations in fluorescence that mean small changes in the intramolecular bonds of the aptamers, confirming the low influence on the affinity of aptamers in this temperature range. In silico analysis also helps to justify these observations. An analysis of the secondary structure of the aptamer for 37 °C (Fig. 6C) reveals that there is significant denaturation of the intramolecular bonds, while for 4 °C most of the structural motifs are maintained, which explains the maintenance of affinity at low incubation temperature. Temperature may have different effects on the aptamers’ performance and depends very much on the conditions under which they were selected and on their sequences. Increased affinity with decreasing temperature has also been observed in other aptamers, which seems to be a result of increased stability of the aptamer structure^56^. Aptamers selected for in vivo applications are normally selected at 37 °C, so its affinity is usually functional over a wider temperature range^29^. Still, extreme temperatures should significantly affect the performance of the aptamers by destroying their functional three-dimensional structure (Fig. 6C). Because of that, the future development of any methodology with this aptamer needs to consider the effect of temperature.

Seen as a promising alternative to antibodies due to the numerous advantages already identified, the selected aptamers were tested in an LFA for the detection of SEA samples. A sandwich format was selected in which aptamers immobilized in the test zone of the strip and aptamers functionalized with AuNPs serve as toxin recognition molecules (Fig. 7). Just as a proof of concept, a mixture containing 100 ng of SEA was prepared, since this is the minimum amount believed to cause intoxication in humans. After incubation in BB with 0.01% tween-20, the sample was applied and flowed onto the assembled lateral flow strip, where it reacted in the test zone revealing a positive response for the presence of SEA. The non-visualization of AuNPs accumulating in the test zone of mixtures containing BSA, one of the most abundant protein in milk (food matrix often associated with SFP), and BB alone shows that the selected aptamers did not interact with these molecules. This simple test proves the functionality of the selected aptamers in detecting SEA, being an alternative with quick, easy and economically cheap synthesis. The lateral flow strip set up for this purpose was based on other ongoing work in our research group, so signal optimization is still needed, as well as the determination of parameters such as detection limit, specificity and sensitivity of the LFA.

In summary, we have successfully demonstrated the selection of DNA aptamers for SEA using a simple, fast and effective SELEX methodology that resorts to magnetic beads, qPCR monitoring, NGS and a bioinformatic tools. Together these tools increase (and facilitate) the odds to identify aptamers with high affinity to the target, while Apt5 demonstrated good affinity within nanomolar range. The aptamers identified shows a promising alternative for the development of diagnostic methodologies against SEA, in particular, aptasensors. LFAs are a simple, fast and cost-effective assay format with high suitability for point-of-care detection of analytes, in which aptamers have demonstrated great applicability in the detection of various molecules, such as proteins and whole cells^57^, but also small compounds such as toxins^14,58^. The findings highlight the potential of aptamers as tailored tools for food safety-based approaches, but also shows their dependency on the environmental conditions.

## Methods

### Materials and instruments

General chemicals for preparing buffers and solutions were purchased from VWR International, Germany. Solutions were prepared with ultra-pure water (H_2_O) from a Millipore water purification system. Biotinylated toxins were obtained from Toxin Technologies (USA). Streptavidin Magnetic Beads and Lambda exonuclease were purchased from New England Biolabs (NEB, UK). Initial ssDNA library and primers were chemically synthesized and HPLC-purified by Eurofins Genomics (Germany). PCR components including NZYSupreme qPCR Green Master Mix (2x) and NZYGelpure purification kit were purchased from NZYTech (Portugal). All PCR reactions were run on a CFX96™ Real-Time PCR Detection System (BioRad, Germany). Quantification of dsDNA was performed using an Eppendorf BioPhotometer (Eppendorf, Germany).

### ssDNA library and primers

The synthetic ssDNA library consisted of a central 40-nt randomized region flanked on either side with fixed primer binding regions for PCR (5′- AGG CCA ACT GGA TAG CGA A-N(40)-CGA ATT CAG CAC TAC CTT TTG GCA AAC GCT AAT AAG GG -3′). Unmodified forward primer (5′- AGG CCA ACT GGA TAG CGA A-3′), unmodified reverse primer (5′- CCC TTA TTA GCG TTT GCC AAA AGG TAG TGC TGA ATT CG -3′) and phosphorylated reverse primer (5′-Phos- CCC TTA TTA GCG TTT GCC AAA AGG TAG TGC TGA ATT CG -3′) were used during amplification steps. The synthetic ssDNA library and the primers were dissolved in H_2_O to a final concentration of 100 µM and 20 µM, respectively.

### Immobilization of SEA on streptavidin-coated magnetic beads

Streptavidin Magnetic Beads (NEB) were used as matrix for immobilization of SEA. Before the immobilization protocol, the desired volume of streptavidin-coated beads at 10 mg (≈ 6–7 × 108) of magnetic beads/mL was transferred to a 1.5 mL low binding tube and washed once with phosphate-buffered saline (PBS), pH 7.4. For the immobilization of the toxin, the washed beads were recovered by magnetic separation and resuspended in the same volume as the initially taken beads with a biotinylated SEA solution. The concentration of SEA was twice the binding capacity of the biotinylated peptides (≈ 200 pmol-mg-1 beads) to ensure the streptavidin saturation. The mixture was incubated for 30 min at room temperature (≈25 °C) with gentle rotation of the tube. After incubation, the tube was placed in a magnetic separator for 2 min and the supernatant discarded (but saved for further analysis). The coated beads were then washed 3 times with PBS, pH 7.4, containing 0.01% [w/v] BSA. Finally, SEA-coated beads were resuspended to 10 mg/mL in BB. The binding efficiency was determined by the difference in SEA concentration used initially and the SEA concentration discarded after incubation, using the Micro BCA Protein Assay Quantification Kit (Thermo Fisher Scientific).

### In vitro selection of DNA aptamers using optSELEX

In total, ten SELEX rounds were performed (Fig. 1). For the first round, 2 nmol of the random ssDNA library (≈ 10^15^ molecules) were prepared in 250 µL BB (138 mM NaCl, Tris-HCl pH 7.4, 2.7 mM KCl, 1 mM CaCl2, 0.5 mM MgCl2), denatured for 5 min at 95 °C, snap cooled on ice for 10 min and then kept at RT for 15 min to allow the formation of folded DNA structures. The folded ssDNA library was then mixed and incubated in a 1.5 mL low binding tube with 0.2 mg (≈ 10^7^ beads) of SEA-coated magnetic beads in a final reaction volume of 250 µL of BB at RT for 30 min with moderate shaking at 550 rpm. After incubation, the binding complexes were collected by magnetic separation, the supernatant containing unbound sequences was discarded and the beads washed once with 1 mL of BB. The binding complexes were then resuspended in 100 μL of H_2_O and the bound sequences were eluted by incubating at 95 °C for 10 min in a thermoblock. The beads were quickly magnetically separated and the supernatant with eluted sequences saved.

In the first SELEX round, the entire pool of ssDNA eluted (100 µL) was subject to PCR (20 reactions, 5 µL ssDNA template each reaction). For this, 50 μL reactions containing 1 × Supreme NZYTaq II 2 × Colourless Master Mix (NZYTech), 0.5 µM forward primer, 0.5 µM phosphorylated-reverse primer, and 5 µL of ssDNA template were prepared. PCR reactions were performed according to the following thermal cycling conditions: initial denaturation step at 95 °C for 5 min, 30 cycles of 94 °C for 30 s, annealing at 53 °C for 30 s and elongation at 72 °C for 30 s, followed by a final extension at 72 °C for 5 min. To recover the ssDNA pool for subsequent selection rounds, PCR products were rendered single-stranded by Lambda-Exonuclease digestion. To that end, all amplification reactions were pooled, and PCR products were separated from primers and other reaction components using the NZYGelpure kit (NZYTech). Purified dsDNA was then quantified using an Eppendorf BioPhotometer (Eppendorf) to estimate the amount of µg of dsDNA. Several reactions up to five µg of purified dsDNA per mixture were prepared with 5 U of Lambda Exonuclease (New England Biolabs) in a total reaction volume of 50 µL at 37 °C for 30 min. The reactions were terminated by adding EDTA (10 mM) and heat inactivation at 75 °C for 10 min. All digestion reactions were pooled, clean-up and the concentration of ssDNA was performed with the NZYGelpure kit (NZYTech). The recovered ssDNA was quantified using an Eppendorf BioPhotometer (Eppendorf) to start the next round.

For the next rounds, after binding reaction and prior to PCR, the number of PCR cycles was optimized to avoid by-product formation during the amplification step as follows: a real-time-PCR reaction was performed, in the same conditions as mentioned above using 1 × NZYSupreme qPCR Green Master Mix, 0.5 µM forward primer, 0.5 µM reverse primer, and 1 µL of ssDNA template. The cycle number before the amplification curve reached the SYBR green fluorescence maximum was then used for subsequent PCR amplification of eluted ssDNA (typically between 9 and 12 cycles, Fig. 2a). Thus, the ssDNA pool was subject to PCR (15 reactions, 2.5 µL ssDNA template each reaction). The remaining ssDNA eluted volume was stored at − 20 °C as a backup. For this, 25 μL reactions containing 1 × Supreme NZYTaq II 2 × Colourless Master Mix (12.5 μL), 0.5 µM forward primer, 0.5 µM phosphorylated-reverse primer, and 2.5 µL of ssDNA template were prepared. All PCR reactions were run on the same conditions mentioned before but using the optimized number of cycles.

Subsequent selection rounds were also performed with decreasing amounts of input ssDNA, decreasing the incubation time, increasing number of washing cycles (from one to three washing cycles) and the addition of competitors, i.e., 0.5 µg/µL BSA, in the washing buffer. To eliminate non-specific binding sequences, negative-selections in rounds R04 to R06 was also performed. For that, of non-coated streptavidin beads (≈ 1 × 10^7^) were added to the ssDNA pool and incubated at RT for 30 min before the real selection reaction. The unbound sequences were then collected by magnetic separation and used to start the next round with SEA-coated beads. The SELEX conditions used to select DNA aptamer for SEA are summarized in Table 1.

### Next-generation sequencing (NGS) and data analysis

The ssDNA pool with the highest binding affinity (round 8) was amplified by PCR (15 reactions, 2.5 µL ssDNA template in each reaction). For this, 25 μL reactions containing 1 × Supreme NZYTaq II 2 × Colourless Master Mix (12.5 μL), 0.5 µM forward primer, 0.5 µM reverse primer, and 2.5 µL of ssDNA template were prepared. All PCR reactions were run on the same conditions mentioned before but using the optimized number of cycles for this round pool. All amplification reactions were pooled, and PCR products were separated from primers and other reaction components using the NZYGelpure kit (NZYTech). Purified dsDNA was then quantified using an Eppendorf BioPhotometer (Eppendorf) and sent for next-generation sequencing (NGS) analysis using adaptor ligation protocol prepared without molecular tags by Genome Sequencer Illumina NovaSeq (Eurofins Genomics Europe Sequencing, Germany).

Afterwards, the raw data files of the sequencing results were introduced into the AptaSUITE software^27^, an automatic platform of multiple algorithms designed for the identification of aptamer candidate sequences and the analysis of the SELEX sequencing results. The primer regions used as well as a 40th central region was predefined in the analysis of the sequencing results. The most frequent oligonucleotide sequences from the top 5 clusters were selected for further evaluation. The free energy (∆G) and secondary structure were calculated using the mfold software^28^. The conditions used for the structure predictions were set according to the SELEX conditions and BB composition: 25 °C, 128 mM Na^+^ and 0.5 mM Mg^2+^.

### Binding assays

The binding assays for the ssDNA pool of rounds 2, 4, 6, 8 and 10 (R02, R04, R06, R08 and R10) and selected individual aptamers (top 5 sequences) were performed using a single standardized concentration under conditions like those of the selection. Briefly, the ssDNA pool/aptamer was prepared in BB at 125 nM, denatured for 5 min at 95 °C, snap cooled on ice for 10 min and then kept at RT for 15 min to allow the formation of folded DNA structures. The folded ssDNA sequences were then mixed and incubated in a 1.5 mL low binding tube with 0.2 mg (≈ 10^7^ beads) of SEA-coated magnetic beads in a final reaction volume of 100 µL of BB at RT for 30 min with moderate shaking at 550 rpm. After incubation, the binding complexes were collected by magnetic separation, the supernatant containing unbound sequences was discarded and the beads washed three times with 1 mL of BB containing 0.5 µg µL^−1^ BSA. The magnetic beads were then resuspended in 100 μL of H_2_O and the bound sequences were eluted by incubating at 95 °C for 10 min in a thermoblock. The beads were quickly magnetically separated and the supernatant with eluted sequences saved. The amount of ssDNA eluted was determined by real-time qPCR. For this, 20 μL reactions containing 1 × NZYSupreme qPCR Green Master Mix, 0.4 μM forward primer, 0.4 μM reverse primer, and 1 μL of ssDNA template were prepared. PCR conditions: Initial denaturation step at 95 °C for 2 min, 25 cycles of 95 °C for 5 s, annealing at 60 °C for 15 s. At the end of each cycle, a SBYR green read was performed. All amplification reactions were performed in triplicates, including a no-template control (NTC) in each run to check for contamination. For each qPCR assay, standard concentrations of the ssDNA library/aptamer under study were also prepared and quantified using the same PCR conditions and a standard curve was build using quantification cycle (Cq) values vs. log (standard concentration) to determine the equation of the line that fits the distribution obtained. At the end, the equation of the linear regression was used to determine the concentration of eluted aptamer and estimate the binding affinity for each ssDNA pool/sequence.

### Determination of the dissociation constant (K_D_)

The affinity of the best aptamer for SEA was determined by performing binding assays but with increasing concentrations of aptamer and a constant amount of SEA-coated magnetic beads (≈10^7^ beads) for each assay. For this, several binding reactions containing increasing concentration of ssDNA aptamer in the range of 1–400 nM were prepared and incubated using the same conditions described above. After incubation, the concentration of SEA-bound aptamers was determined, after washing and elution steps, by qPCR quantification as described for binding assays. Three biological replicates were performed for each aptamer concentration. The K_D_ was determined by a non-linear regression analysis using SigmaPlot version 12.5, from Systat Software, Inc., USA.

### Effects of temperature on aptamer binding affinity

To ascertain the effect of temperature and pH on aptamer binding affinity, the selected aptamer was incubated with SEA (≈ 10^7^ coated beads) under the same conditions as for the binding assays but with the following variations: (1) BB, pH 7.4, at 4 °C, and (2) BB, pH 7.4, at 37 °C. A binding reaction under SELEX conditions was also performed as a control. After incubation, the concentration of SEA-bound aptamers was determined by qPCR quantification as described for binding assays.

### Aptamer-based lateral flow assay

LFA were prepared according to the schematic shown in Fig. 7. These included the sample pad with 1.5 cm, glass fibber membrane, grade 8950 (Ahlstrom Munksjo, Finland), the test zone with 2.5 cm, nitrocellulose membrane (FF170HP, Whatman, United Kingdom) and the absorbent pad with 2 cm, cellulose membrane, grade 270 (Ahlstrom Munksjo), all supported by a backing card (KN-2211, Kenosha, Netherlands) with 5 mm width.

The lateral flow assay was designed in a sandwich format, in which aptamer functionalized gold nanoparticles (AuNPs) were used as visual recognition elements in solution and biotinylated aptamers as immobilized recognition elements in test zone. To this end, Apt5 was covalently attached to 15 nm AuNPs (OD50) via the 3′ end (Nanopartz, United States). A polyT tail was added to the 3′ end of the aptamer as a sphere surface spacer. Biotinylated Apt1, Apt2, Apt3 and Apt4 were also synthesized based on biotin-TEG modification at 5′ end (Eurogentec, Belgium).

For the test line, a pool containing 140 pmol of each biotinylated aptamer (Apt1, Apt2, Apt3 and Apt4) were incubated with a streptavidin solution (Alfa Aesar, United States) in PBS, ensuring a 1:4 streptavidin:biotin ratio, for 2 h at RT. After that, the reactional mixtures (1 μL) were dispersed on the nitrocellulose membrane (test zone). A DNA probe (140 pmol–5′ CCC TTA TTA GCG TTT GCC AAA AGG TAG TGC TGA ATT CG – 3′) complementary to Apt5 was also incubated with a streptavidin and then immobilized on the nitrocellulose membrane as a test control. The strips were dried at 37 °C for 30 min and then stored at 4 °C until use.

Sample containing 50 μL of a SEA solution (2 ng/µL) as well as negative samples (50 μL of 1 mg/mL BSA and 50 μL of BB) were prepared and incubated with Apt5-AuNPs (OD3) in 1 × BB with 0.01% tween-20 in total volume of 100 μL for 10 min at RT. The samples were applied to the sample pad, allowing the solution to flow on the strip until the test lines were visualized by the accumulation of AuNPs.

## Data Availability

The datasets generated and/or analysed during the current study are available in the NCBI repository and can be downloaded using the accession number PRJNA916129.
